# Reducing Hinge Flexibility of CAR-T Cells Prolongs Survival *In Vivo* With Low Cytokines Release

**DOI:** 10.3389/fimmu.2021.724211

**Published:** 2021-10-05

**Authors:** Ang Zhang, Yao Sun, Jie Du, Yansheng Dong, Honggang Pang, Lei Ma, Shaoyan Si, Zhong Zhang, Mingyi He, Yang Yue, Xiaoli Zhang, Weichao Zhao, Jianjun Pi, Mindong Chang, Quanjun Wang, Yikun Zhang

**Affiliations:** ^1^ Department of Hematology, Strategic Support Force Medical Center, Beijing, China; ^2^ The Department of Hematology, Beijing, China; ^3^ Department of Hematology, Fifth Medical Center of Chinese PLA General Hospital, Beijing, China; ^4^ SAFE Pharmaceutical Research Institute Co. Ltd, HeBei, China; ^5^ Department of Emergency, Affiliated Zhongshan Hospital, Dalian University, Dalian, China; ^6^ Academy of Military Medical Sciences, Academy of Military Sciences, Beijing, China; ^7^ Comprehensive Basic Experiment, Beijing, China; ^8^ The Department of Cardiovascular Medicine, Beijing, China; ^9^ The Department of Respiratory Medicine, Beijing, China; ^10^ Strategic Support Force Medical Center, The Department of Surgical Oncology, The First Affiliated Hospital of Xi’an Jiaotong University, Xi’an, China; ^11^ National Beijing Center for Drug Safety Evaluation and Research, State Key Laboratory of Medical Countermeasures and Toxicology, Institute of Pharmacology and Toxicology, Academy of Military Sciences, Beijing, China

**Keywords:** chimeric antigen receptor (CAR T), hinge region, cytokine release storm (CRS), structure optimization, cellular immunotherapy, gene modified T cell

## Abstract

Chimeric antigen receptor (CAR)-modified T cells targeting CD19 demonstrate unparalleled responses in B cell malignancies. However, high tumor burden limits clinical efficacy and increases the risk of cytokine release syndrome and neurotoxicity, which is associated with over-activation of the CAR-T cells. The hinge domain plays an important role in the function of CAR-T cells. We hypothesized that deletion of glycine, an amino acid with good flexibility, may reduce the flexibility of the hinge region, thereby mitigating CAR-T cell over-activation. This study involved generating a novel CAR by deletion of two consecutive glycine residues in the CD8 hinge domain of second-generation (2nd) CAR, thereafter named 2nd-GG CAR. The 2nd-GG CAR-T cells showed similar efficacy of CAR expression but lower hinge flexibility, and its protein affinity to CD19 protein was lower than that of 2nd CAR-T cells. Compared to the 2nd CAR-T cells, 2nd-GG CAR-T cells reduced proinflammatory cytokine secretion without diminishing the specific cytotoxicity toward tumor cells *in vitro*. Furthermore, 2nd-GG CAR-T cells prolonged overall survival in an immunodeficient mouse model bearing NALM-6 when tumor burden was high. This study demonstrated that a lower-flexibility of CD8α hinge improved survival under high tumor burden and reduced proinflammatory cytokines in preclinical studies. While there is potential for improved safety and efficacy, yet this needs validation with clinical trials.

## Introduction

Chimeric antigen receptor T cell (CAR-T) therapy for hematological malignancies has demonstrated tremendous clinical outcomes (1, 2). Four CAR-T cell products have been approved globally, including Kite’s Yescarta and Tecartus, Novartis’s Kymriah, and BMS’s Breyanzi, all targeting CD19 antigen (2–4). However, a high tumor burden often indicates poor prognosis and significant adverse reactions after CAR-T therapy, which may be related to the over-activation of CAR-T cells (5–8). Therefore, patients with a high tumor burden have an unmet medical need for anti-CD19 CAR-T therapy.

Investigators are currently striving to improve the safety and efficacy of CAR-T cells by optimizing CAR designs to overcome their existing limitations (9). These include cytokine release syndrome (CRS) and immune-effector cell associated neurotoxicity syndrome (ICANS), both related to the excessive release of cytokines and limited persistence caused by activation-induced cell death (AICD) (10–15). The standard CAR design consists of four modular components: the antigen binding domain, hinge domain, transmembrane domain, and intracellular signaling domain, each of which has a specific function and thus the potential to be optimized (16). More attention has been paid to the improvement of signal regions, including antigen recognition and signaling argument regions such as the costimulatory domain and immunoreceptor tyrosine-based activation motif (ITAM) of CD3ξ (17, 18).

In recent years, a growing number of studies have demonstrated the significant function of non-signaling regions. The properties of the hinge and transmembrane domains also influence CAR-T cell cytokine production and AICD (19), which are related to the anti-tumor efficacy and the loss of CAR, respectively (20, 21). Ying et al. (22) constructed a new CAR design with longer extracellular and intracellular domains named CD19-BBz (86) CAR T cells, which produced a potent and durable anti-lymphoma response without causing neurotoxicity or severe CRS (greater than grade 1). The hinge provides sufficient flexibility to overcome steric hindrance, and length to facilitate access to the target antigen (23). It thus seems reasonable to down-regulate the activation of CAR-T cells by reducing the flexibility of the hinge region, thereby improving efficacy and safety. Glycine, the smallest amino acid is unique because unlike all others, it contains hydrogen as its side chain rather than a carbon (24), permitting much more conformational flexibility. (Gly_4_Ser)_n_ is often used as a linker for different polypeptides because it is not prone to misfolding errors, and Gly plays an irreplaceable role in this structure (25).

Consequently, this study entailed designing a novel CAR by deleting two consecutive glycine residues in the CD8 hinge domain of traditional second-generation (2nd) CAR and named the FMC63-CD8(Gly2-deletion)-4-1BB-CD3ζ CAR as 2nd-GG CAR. Studies were then conducted to verify the flexibility and affinity of this new CAR, and compare the functions of 2nd and 2nd-GG CAR-T cells *in vitro* and *in vivo*.

## Materials and Methods

### Cell Lines and Cell Culture Conditions

Cell lines were cultured according to the manufacturers’ recommendations. NALM-6 is a pre-B cell acute lymphoblastic leukemia (ALL) cell line with high expression of CD19 (German DSMZ cell collection Cat#: ACC128). NALM-6-GFP-luciferase (luc) is a stable cell line engineered to express GFP-luciferase. K562 is a chronic myelogenous leukemia cell line (ATCC; Cat#: CCL-243). K562-CD19 and K562-CD19-GFP are stable cell lines engineered to express CD19 and/or GFP. 786o is a renal cell adenocarcinoma cell line (ATCC; Cat#: CRL-1932™). CD19 was transduced using a lentivirus system into 786o to produce 786o-CD19. The method of tumor cells culture refers to our previous study (26).

### Generation of CAR Constructs

Generation of lentiviral constructs and production of lentiviral particles refer to our previous study (27). The conventional second-generation 2nd CAR was constructed by the fusion of CD19 scFv, CD8 hinge and transmembrane, 4-1BB, and CD3ζ. The structure of 2nd-GG is same to the 2nd CAR except for deletion of two consecutive glycine in the CD8 hinge. Nucleotide sequence of CD8 hinge in 2nd-CAR and 2nd-GG CAR are shown in **Supplementary Figure 2**. 

### Selection, Activation, and Lentivector Transduction of CD3+ T Cells

Blood samples from healthy volunteers were obtained using an approved protocol by the Ethics Committee of the Fifth Medical Center of Chinese PLA General Hospital (Ethical code: Ky-2018-5-37). These studies were conducted following the Declaration of Helsinki. All subjects provided written informed consent before participation in the present study. The methods of T cell isolation and culture and gene transfer refer to our previous study (26).

### Binding Assay

Briefly, through the measurement of the fluorescence intensity of different CAR T cells to CD19 protein at various concentrations, their affinity for CD19 protein can be determined. Specifically, mock-T, 2nd CAR-T, and 2nd-GG CAR-T cells were washed twice by centrifugation with PBS (1% BSA). They were treated with CD19-Fc protein (11880- H02H) at final concentrations of 180 µg/mL, 72 µg/mL, 28.8 µg/mL, 11.52 µg/mL, 4.61 µg/mL, 1.84 µg/mL, 0.74 µg/mL, 0.29 µg/mL, 0.12 µg/mL, or 0.05 µg/mL, incubated at 4°C in darkness for 45 min, and washed twice with a PBS washing solution by centrifugation. Next, the cells were treated with 10 µL goat anti-human IgG (FC)/FITC, incubated at 4°C in darkness for 20 min, washed twice with a washing solution by centrifugation, and tested utilizing flow cytometry (NovoCyte D3010).

### Cytotoxicity Assay

Briefly, CFSE-labeled targets were incubated at the indicated ratios with effector T cells for 12–16 h or 6–8 h. The cells were then harvested, and Annexin V and 7-AAD were added prior to flow cytometric analysis. The residual live target cells were CFSE+ Annexin V- 7-AAD-. E:T ratios designated the ratios of the absolute number of CAR T cells to target cells. The number of T cells was the same as that in the 2nd CAR group. All experiments were carried out in triplicate.

### Cytokine Production

Effector cells (5 × 10^4^) and target cells (5 × 10^4^) were incubated at a 1:1 ratio in RPMI (10% FBS) media with 10% human serum for 24 h. Cytokine concentration in the culture supernatant and mouse serum was measured with enzyme-linked immunosorbent assay (ELISA) kits (MultiSciences Biotech Co., Ltd., China) for human IFN-γ, TNF-α, and IL-2. E:T ratio designated the ratio of the absolute number of CAR T cells to target cells. The number of T cells was the same as that in the 2nd CAR group.

### Flow Cytometry

Anti-human antibodies were purchased from Becton Dickinson, BioLegend, and Miltenyi Biotec. The Accuri C6 (Becton Dickinson, USA), FACS Calibur (Becton Dickinson, USA), and BD FACSAriaTM II cell sorter were used for the analysis of various samples. Anti-human antibodies were purchased from BioLegend, eBioscience, Acrobiosystems, or BD. Cells were isolated from *in vitro* cultures or from animals, washed once with PBS supplemented with 2% FCS, and stained on ice after blocking Fc receptors. In all analyses, the population of interest was gated based on forward vs. side scatter characteristics followed by singlet gating.

### Mouse Xenograft Tumor Model

Animal experiments were conducted at the National Beijing Center for Drug Safety Evaluation and Research and at the SAFE Pharmaceutical Research Institute Co.,Ltd (IACUC-2019-001). Female NSG mice (28) aged 6–8 weeks were used. For NALM-6-acute precursor B-ALL models, 10^6^ tumor cells were intravenously injected with PBS, and tumors were measured by the total bioluminescent flux using a Xenogen Imaging System (PerkinElmer-IVIS Lumina III). Peripheral blood was collected *via* the submandibular vein.

### Statistical Analysis

Statistical analyses were performed using Prism version 7.0 (GraphPad). For studies comparing two groups, we utilized a Students t-test. Log rank (Mantel Cox) test was used to analyze *in vivo* survival. Survival curves were constructed using Kaplan–Meier methodology.

## Results

### Deletion of Gly-Gly in CD8 Hinge Region of CAR Reduced the Flexibility of Hinge Without Affecting the CAR Expression Efficiency

**Figure 1:**
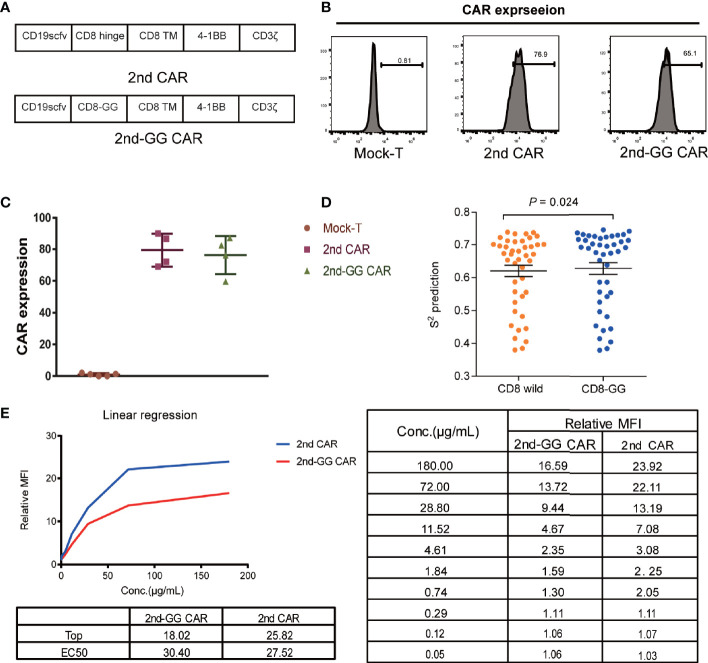
Schematic diagram and expression efficiency of 2nd and 2nd-GG CAR-T cells. **(A)** Diagrammatic model of 2nd and 2nd-GG CAR. Schematic of CAR containing scfv (FMC63), variations in the hinge, extra-membrane, and transmembrane domains. The hinge region of 2nd-GG deleted two Gly compared with that of the 2nd CAR, and the rest of the sequences were the same. **(B)** Typical flow cytometry detection of the expression efficiency of 2nd and 2nd-GG CAR on T cells. **(C)** Expression efficiency of 2nd and 2nd-GG on T cells 5-6 days after culture *in vitro* determined by flow cytometry (mean ± SD, n = 5). T cells are derived from at least three different healthy donors. **(D)**. Comparison of the flexibility between the CD8 hinge and the CD8-GG hinge. S2 order parameter (S2 RCI) values were estimated from chemical shift values using the Random Coil Index (RCI) software. S2 is inversely proportional to the hinge region flexibility. **(E)**. The affinity of CD19 protein to different CAR T cells: 2nd CAR-T cells > 2nd-GG CAR-T cells. The EC50 of 2nd and 2nd-GG CAR-T cells binding to CD19 protein was determined by flow cytometry. EC50, 50% maximal effective concentration. CAR, chimeric antigen receptor; FITC, fluorescein isothiocyanate.

The 2nd CAR-T cells, structured as FMC63-CD8-4-1BB-CD3ζ, have shown promising efficacy in clinical studies (1). To decrease the flexibility of the hinge region, deletion mutations were performed on two consecutive Glys in the wild-type CD8 hinge region of FMC63-CD8-4-1BB-CD3ζ CAR, and this novel CAR was named 2nd-GG CAR (**Figure 1A**). The specific nucleic acid sequences of the wild CD8 hinge region and the CD8 hinge region with deletion of 2 Gly are shown in **Supplement Figure 1**. The transduction efficiency of 2nd CAR and 2nd-GG CAR on human T cells was similar (approximately 70%) (**Figures 1B, C**). The S2 order parameters represent the restriction of movement of an atomic bond vector with respect to the molecular reference frame. The greater the value of S2, the less flexible the protein. Thus, the flexibility of the CD8-GG hinge region was less than that of the CD8 hinge region according to the index of S2 from DynaMine (29) (**Figure 1D**). Furthermore, when the two CAR-T cells were individually incubated with different concentrations of CD19 protein, the 2nd-GG CAR-T cells showed weaker binding ability to CD19 protein than 2nd CAR-T cells (**Figure 1E**).

### 2nd-GG CAR-T Cells Showed Similar Killing Efficiency but Secreted Less Proinflammatory Cytokines Compared to 2nd-GG CAR-T Cells *In Vitro*

**Figure 2:**
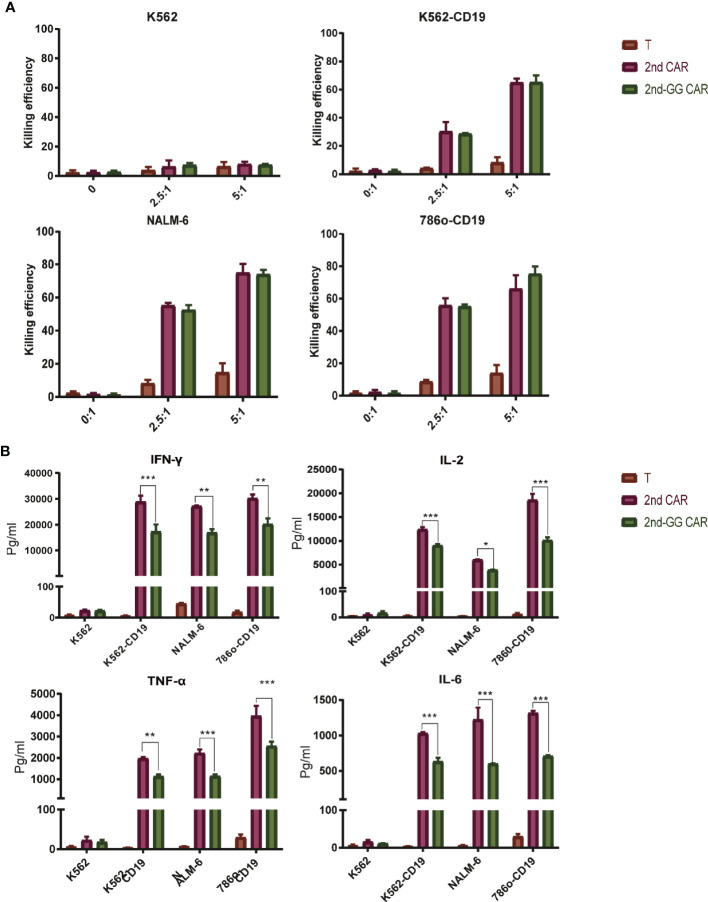
The killing efficiency and cytokine secretion of 2nd CAR-T and 2nd-GG CAR-T cells towards tumor cells. **(A)** Cytotoxic percentages of targeted cells by mock T, 2nd and 2nd-GG CAR-T cells after 8–10 h of co-culture *in vitro*. E: T (2.5:1 and 5:1) designate the ratios of the absolute number of CAR T cells to target cells, specifically K562, NALM-6, 786o-CD19, and K562-CD19. The number of mock T cells is the same as in the 2nd CAR-T cells group. Results are representative of at least three independent experiments with T cells from different healthy donors. **(B)** Human IFNγ, TNF-α, IL-2 and IL-6 production by mock T, 2nd and 2nd-GG CAR-T cells. Cytokine concentrations in the media were measured after 24 h of co-incubation with different target cells at E: T of 1:1. Values are mean ± SD of triplicate specimens obtained with T cells isolated from one healthy donor. *P < 0.05; **P < 0.01; ***P < 0.005.

To evaluate the effector function of the two different CAR-T cells, a killing (cytotoxicity) and cytokine secretion assays were conducted on different cell lines. These were: NALM-6, a precursor B-cell leukemia cell line that naturally expresses CD19, plus the 786o and K562 cell lines which are CD19 negative (**Figure 2**). The two CAR-T cells showed similar cytotoxic efficacy against the CD19-positive and negative cell lines, with no statistically significant differences.

It is well known that cytokines secreted from CAR-T cells trigger an overactivation of the immune system, ultimately leading to CRS (30). We therefore examined the pro-inflammatory factors released after the incubation of CAR T cells with different tumor cells. Following incubation with CD19+ target cells, the amount of proinflammatory cytokines secreted by 2nd-GG CAR-T cells was less than that of 2nd-GG CAR-T cells (P<0.01). None of the CAR-T cells produced specific killing effects or proinflammatory factors against K562, a CD19- tumor cell line, demonstrating the antigen-specificity towards CD19 by the 2nd-GG CAR-T cells.

### 2nd-GG CAR-T Cells Exhibited Similar Antitumor Efficacy but Less Proinflammatory Cytokines Release in Mouse Model With Moderate Tumor Burden

**Figure 3:**
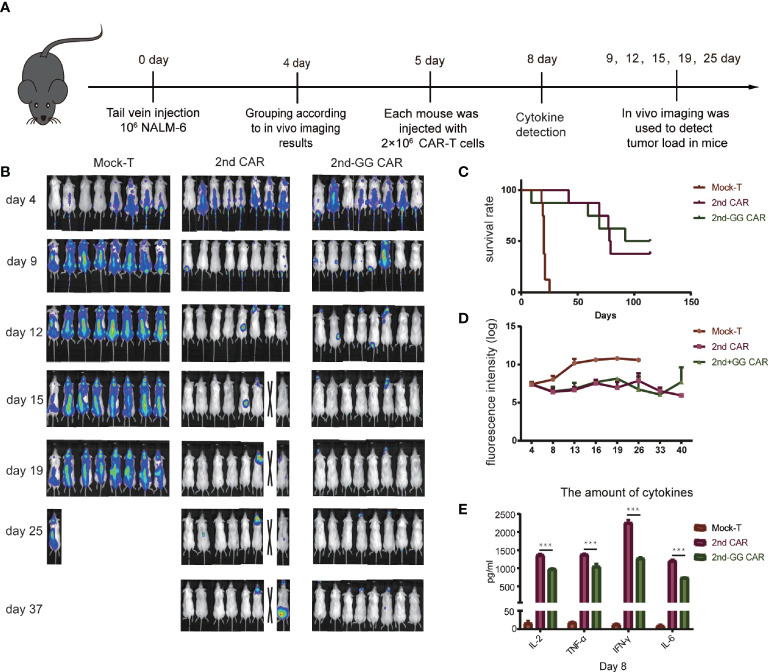
The antitumor efficacy and cytokines release of different CAR-T cells in moderate tumor load models. **(A)** Diagrammatic representations of the experimental procedures. **(B)** Representative bioluminescent images are shown. **(C)** Overall survival curves of NALM-6 -GFP-luc challenged mice (n = 8). **(D)** Tumor burden-total flux (log) for each mouse was quantified and averaged by group. (mean ± SEM) **(E)** On day 8, approximately 1,000 µL of blood were collected from the caudal vein of each mouse mixed to detect the concentration of human IL-2, TNF-α, IFN-γ, and IL-6 using an ELISA-kit. (mean ± SD, n = 2). ***P < 0.005.

Although 2nd-GG CAR-T cells showed a similar specific immune response to CD19+ tumor cells *in vitro* compared with 2nd CAR-T cells, their antitumor efficacy in animal models needs to be further verified. The anti-tumor efficacy of CAR-T cells in NSG immunodeficient mice bearing NALM-6-GFP-luc(luciferase) was subsequently investigated, as detailed in **Figure 3A**. Both 2nd-GG and 2nd CAR-T cells exhibited improved overall survival (OS) and reduced tumor burden compared with the mock-T cells, demonstrating improved tumor control of both CAR-T cells (**Figures 3B, D**). Furthermore, compared to the 2nd CAR-T cell group, the OS in those administered 2nd-GG CAR-T cells was prolonged, although there was no statistical difference, as shown in **Figure 3C**. As expected, 2nd-GG CAR-T cells secreted less human proinflammatory cytokines, particularly IL-6 and IFN-γ, compared to the 2nd CAR-T cells *in vivo* (**Figure 3E**). In order to distinguish it from the following experiment with a higher tumor burden, this experiment was referred to as “with moderate tumor load”. The 2nd-GG CAR-T cells did not show sufficient advantage compared to the 2nd CAR-T cells in experiments with moderate tumor burden, owing to the relatively lower tumor load.

### 2nd-GG CAR-T Cells Significantly Improved Antitumor Activity in Mouse Model With High Tumor Burden

**Figure 4:**
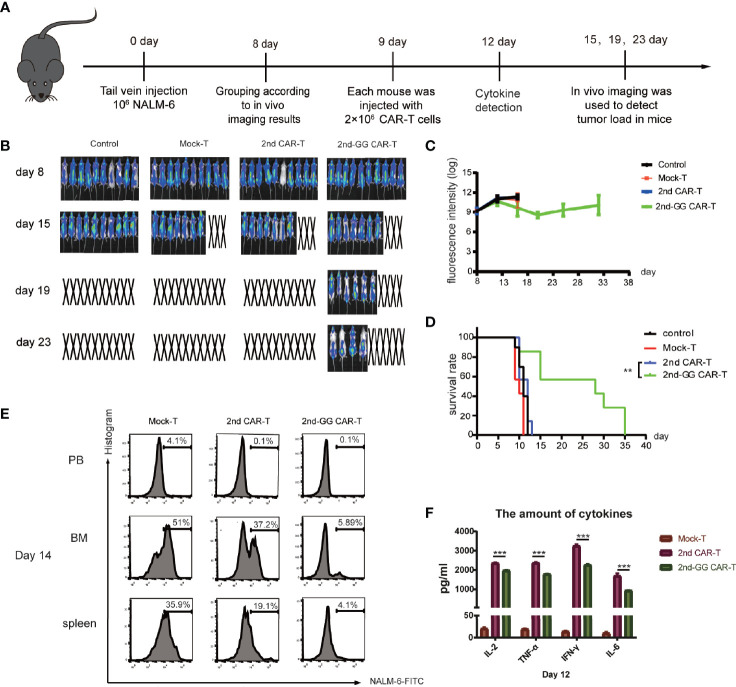
The antitumor efficacy and cytokines release of different CAR-T cells in high tumor load models. **(A)** Diagrammatic representations of the experimental procedures. **(B)** Representative bioluminescent images are shown. **(C)** Overall survival curves of NALM-6 -GFP-luc challenged mice (n = 8). **(D)** Tumor burden-total flux (log) for each mouse was quantified and averaged by group. (mean ± SEM) **(E)** On day 14, one mouse was randomly euthanized from the Mock-T, 2nd CAR-T and 2nd-GG CAR-T groups. Cell suspensions from peripheral blood, bone marrow and spleen were collected and ground for flow cytometry detection. Since the NALM-6 cells were engineered to express GFP, the tumor load was reflected by the expression percentage of GFP+ cells. **(F)** On day 12, approximately 1,000 µL of blood were collected from the caudal vein of each mouse to detect the concentration of human IL-2, TNF-α, IFN-γ, and IL-6 using an ELISA-kit. (mean ± SD, n = 2). **P < 0.01, ***P < 0.005.

A high tumor burden often indicates a poor prognosis and significant adverse reactions after CAR-T therapy (31). It is suggested that a high tumor burden might affect the efficacy of CAR-T cell therapy (32, 33). It was thus hypothesized that CAR T cells behave differently in mouse models with different tumor burdens. To mimic the clinical situation of a high tumor burden, NSG mice bearing NALM6-Luc tumors received delayed CAR-T cell infusion to increase the tumor load. The specific schedule is shown in **Figure 4A**. When NSG mice were challenged with high tumor burden, 2nd-GG CAR-T cells showed significantly improved overall survival compared with 2nd CAR-T cells, while the 2nd CAR-T cells showed no advantage over the mock-T cells (**Figures 4B, D**). The tumor load in group of 2nd-GG CAR-T was lower than that of 2nd CAR T (P>0.05) on day 15 and showed a downward trend (**Figure 4C**). The anergy of 2nd CAR-T cell in the mouse model with high tumor load is likely related to AICD. One mouse from each group was randomly selected on day 14, to evaluate the tumor load of peripheral blood (PB), bone marrow (BM), and spleen by flow cytometry. The results showed that the tumor burden of the 2nd-GG group was less than that of the other two groups after treatment (**Figure 4E**). Similarly, the amount of human proinflammatory cytokines secreted by 2nd-GG CAR T cells was lower than that of 2nd CAR T cells (**Figure 4F**).

Overall, 2nd-GG CAR-T cells exhibited stronger antitumor activity and lower cytokine release in the high tumor burden model than the 2nd CAR-T cells.

## Discussion

This study demonstrated that 2nd-GG CAR exhibits lower flexibility and affinity for the CD19 antigen. The 2nd-GG CAR-T cells produced lower levels of cytokines, yet showed similar cytotoxicity to CD19+ tumor cells as 2nd CAR-T cells *in vitro*. However, 2nd-GG CAR-T cells show lower cytokine release in mouse models with moderate and high tumor burden, and prolong overall survival in animal models with high tumor burden.

Currently, the indication for anti-CD19 CAR T cells has been mainly for relapse and refractory B-cell malignancies, which are often insensitive to traditional radiotherapy and chemotherapy. Furthermore, an inevitable vein-to-vein time interval, typically 3-8 weeks, is required for patients preparing for CAR-T cell therapy. Pivotal trials of approved treatments have resulted in up to a third of the enrolled patients failing to receive the product. It has not been determined if bridging therapy is necessary during this gap, and which treatment regimen may be better (34). Although off-the-shelf cell therapy or Fast CAR-T cells may shorten the vein-to-vein time interval, it is still under clinical study (35). Therefore, the high tumor burden in patients before CAR-T cell therapy is an unavoidable problem. It has been reported that both the efficiency and the incidence of adverse reactions, such as CRS of the anti-CD19 second-generation CAR T cells, increased in patients with high tumor burden (36–38). Many studies have demonstrated that reduced activation of anti-CD19 CAR-T cells improves the safety and efficiency of CAR-T cells (22). This could be achieved through reducing anti-CD19 CAR T cell activation by diminishing scFv affinity (39), increasing the hinge and transmembrane region (22), replacing the co-stimulatory molecule from CD28 to 4-1BB (18), and mutation of the immunoreceptor tyrosine-based activation motif (ITAM) region of CD3ζ (40).

The hinge region has a significant impact on the function of CAR T cells, and its components are often derived from the IgG family or the co-receptor of T cells (CD4/CD8) (41), but the specific mechanism is still unclear (9). Studies have shown that the hinge region provides a spatial location for the recognition of scFv and antigens. When the epitope recognized by CAR is in a membrane proximal position, the hinge region is necessary for the recognition of CAR-T cells by antigens, such as when targeting NCAM or 5T4. Whereas if the epitope recognized by CAR is a membrane distal epitope, the hinge region is negligible for the recognition of CAR-T cells by antigens, such as when targeting CEA (42). In general, little is known about the role of the hinge domain, and strategies to optimize it need to be creatively explored.

The flexibility of the hinge region has been shown to affect the CAR T cell function. The addition of a flexible IgG hinge instead of a CD28 hinge alone (SD28ζ) led to more pro-cytokines produce and better recognition of the MUC1 epitope compared to S28ζ CAR-T cells (43). However, further verification is needed to determine whether reducing the flexibility of the hinge region can decrease CAR-T activity. We removed two consecutive glycine residues in the hinge region to reduce the flexibility of the hinge domain, thus resulting in better tumor control and lower release of inflammatory factors such as TNF-α and IL-6, which are the key molecules triggering the cytokine storm. This can be explained by the fact that reducing the flexibility of the hinge domain prevents overactivation of CAR-T cells, especially under high tumor load. However, the specific mechanism is unknown and warrants further investigation. Although studies have shown that the persistence of CAR-T cells is essential for immune surveillance of tumors, CAR gene copy numbers were unfortunately not measured (6). Studies have shown that the formation of immune synapses by CAR influences the function of CAR-T cells and changes the flexibility of the hinge region (44, 45). This may alter the formation of immune synapses in CAR, thus affecting the function of CAR-T cells, though it needs to be further explored.

Although we observed a downward trend in tumor load in the 2nd-GG group, it is a limitation of our study that the lack of evidence for enhanced anti-tumor activity of 2nd-GG CAR-T *in vivo*. Mice in the group of Mock-T, which had very low level of cytokines, had the highest mortality at day 15. Therefore, the death of mice was not caused by excessive release of cytokines. Recent study demonstrated that patients with high tumor burden had higher immune dysregulation with increased serum inflammatory markers and tumor IFN signaling. IFN signaling is associated with the expression of multiple checkpoint ligands and inferior response to CAR-T therapy (46). Therefore, we considered the direct cause of death in high tumor burden model was the increased tumor load. We hypothesized that lower levels of inflammatory cytokine *in vivo* improved activity of 2nd-GG CAR-T through correct the immune dysregulation and reduce tumor IFN signaling, which requires further detection of phenotypes and exhaustion markers of T cells to confirm.

The present study demonstrated that a novel CD19 CAR with a less flexible hinge domain showed prolonged survival of mice under high tumor burden in preclinical studies. While there is potential for improved safety and efficacy, yet this needs validation with clinical trials.

## Data Availability Statement

All data generated and analyzed for this study are included in the article/**Supplementary Material**.

## Ethics Statement

The studies involving human participants were reviewed and approved by the Ethics committee of Fifth Medical Center of Chinese PLA General Hospital. The patients/participants provided their written informed consent to participate in this study. Written informed consent was obtained from the individual(s) for the publication of any potentially identifiable images or data included in this article The animal study was reviewed and approved by the Ethics committee of Fifth Medical Center of Chinese PLA General Hospital (ky-2018-5-37). Written informed consent was obtained from the owners for the participation of their animals in this study.

## Author Contributions

AZ designed the experiments. AZ and YS wrote the main body of the paper. AZ, YS, JD, YD, and HP performed the experiments and wrote the main body of the paper, with contributions from LM, SS, ZZ, MH, YY, XZ, WZ, and JP. YZ, QW, and MC supervise the experiments and revised the manuscript. All authors contributed to the article and approved the submitted version.

## Funding

This work was supported by a grant from 18-163-12-ZD-013-008-02, 2018ZX09711003-007 and 2018ZX09201017-003.

## Conflict of Interest

Authors JD and YD were employed by SAFE Pharmaceutical Research Institute Co., Ltd.

The remaining authors declare that the research was conducted in the absence of any commercial or financial relationships that could be construed as a potential conflict of interest.

## Publisher’s Note

All claims expressed in this article are solely those of the authors and do not necessarily represent those of their affiliated organizations, or those of the publisher, the editors and the reviewers. Any product that may be evaluated in this article, or claim that may be made by its manufacturer, is not guaranteed or endorsed by the publisher.
