# Supplementary material for: Reducing Hinge Flexibility of CAR-T Cells Prolongs Survival In Vivo With Low Cytokines Release
Source: Front Immunol. 2021 Oct 5;12:724211. doi: 10.3389/fimmu.2021.724211 (PMC8524077; doi:10.3389/fimmu.2021.724211)
Supplement: Supplementary file 1 [file DataSheet_1.pdf]

A. the nucleotide sequence of wild CD8α hinge

ACCACTACCCCAGCACCGAGGCCACCCACCCCGGCTCCTACCATCGCCT
CCCAGCCTCTGTCCCTGCGTCCGGAGGCATGTAGACCCGCAGCTGCCG
TGCATACCCGGGGTCTTGACTTCGCCTGCGATATCTACATTTGGGCCCCT
CTGGCTGGTACTTGCGGGGTCCTGCTGCTTTCACTCGTGATCACTCTTTA
CTGT

B. the nucleotide sequence of CD8α-GG hinge

ACCACTACCCCAGCACCGAGGCCACCCACCCCGGCTCCTACCATCGCCT
CCCAGCCTCTGTCCCTGCGTCCGGAGGCATGTAGACCCGCAGCTGGTG
GGGCCGTGCATACCCGGGGTCTTGACTTCGCCTGCGAT

Nucleotide sequence of the wild-type CD8α hinge region was shown in A and the CD8α-GG was shown in B.
