# Supplementary material for: Reducing Hinge Flexibility of CAR-T Cells Prolongs Survival In Vivo With Low Cytokines Release
Source: Front Immunol. 2021 Oct 5;12:724211. doi: 10.3389/fimmu.2021.724211 (PMC8524077; doi:10.3389/fimmu.2021.724211)
Supplement: Supplementary file 2 [file DataSheet_2.pdf]

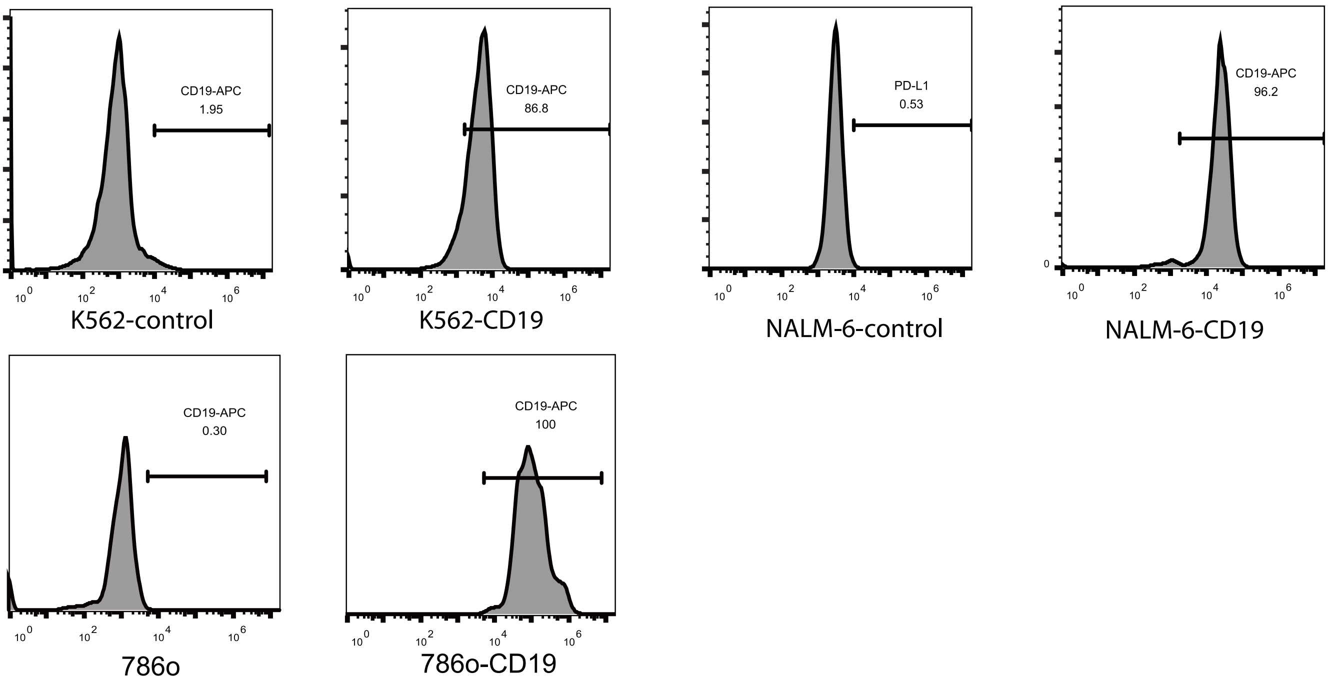

The figure shows the CD19 expression efficiency of overexpression CD19 gene modified K562, 786o and natural NALM-6 cell line.
